# Non-pulsed Sinusoidal Electromagnetic Field Rescues Animals From Severe Ischemic Stroke via NO Activation

**DOI:** 10.3389/fnins.2019.00561

**Published:** 2019-06-19

**Authors:** Lena P. Font, Miriam M. Cardonne, Hannelore Kemps, Raf Meesen, Oneida F. Salmon, Fidel G. González, Ivo Lambrichts, Jean-Michel Rigo, Bert Brône, Annelies Bronckaers

**Affiliations:** ^1^Faculty of Medicine and Life Sciences, BIOMED, UHasselt – Hasselt University, Diepenbeek, Belgium; ^2^Centro Nacional de Electromagnetismo Aplicado, Universidad de Oriente, Santiago de Cuba, Cuba

**Keywords:** cerebral ischemia, nitric oxide, electromagnetic field, endothelial cell, neuroprotection

## Abstract

Despite the high prevalence and devastating outcome, only a few treatment options for cerebral ischemic stroke exist. Based on the nitric oxide (NO)-stimulating capacity of Non-pulsed Sinusoidal Electromagnetic Field (NP-SEMF) and the possible neuroprotective role of NO in ischemic stroke, we hypothesized that NP-SEMF is able to enhance survival and neurological outcome in a rat model of cerebral ischemia. The animals, in which ischemic injury was induced by occlusion of both common carotid arteries, received 20 min of NP-SEMF of either 10 or 60 Hz daily for 4 days. NP-SEMF dramatically increased survival, reduced the size of the infarcted brain area and significantly improved the neurological score of the surviving rats. Corresponding to previous reports, NP-SEMF was able to induce NO production *in vitro*. The importance of NO as a key signaling molecule was highlighted by inhibition of the NP-SEMF beneficial effects in the rat stroke model after blocking NO synthase (NOS). Our results indicate for the first time that NP-SEMF exposure (13.5 mT at 60 and 10 Hz) improves the survival and neurological outcome of rats subjected to cerebral ischemia and that this effect is mediated by NO, underlining the great therapeutic potential of NP-SEMF as a therapy for ischemic stroke.

## Introduction

Cerebral ischemic stroke is caused by the occlusion of a major artery in the brain, which results in extensive brain tissue loss. Cerebral ischemic stroke is a major cause of mortality and disability worldwide (Kissela et al., 2009; Feigin et al., 2014). Currently, the only available treatment options are tissue plasminogen activator and thrombectomy, which have significant effectivity after stroke. However, these therapies can only be used in a small subset of cases, indicating the clear need for new interventions (Zhang et al., 2013).

Nitric oxide (NO) is an important signaling molecule that is synthesized from its precursor L-arginine by nitric oxide synthase (NOS). Following stroke, NO produced by the neuronal and inducible isoforms of NOS exerts neurotoxic effects, while NO supplied by endothelial NOS (eNOS) is beneficial by stimulating several mechanisms such as vasodilation, angiogenesis, and neurogenesis. As a consequence of these later positive effects, NO donors display therapeutic effects in animal stroke models (Terpolilli et al., 2012; Garry et al., 2015). Extremely low frequency electromagnetic field (ELF-MF) has been shown to induce NO production (Jelenkovic et al., 2006; Bragin et al., 2015). ELF-MF is defined by a magnetic flux below 20 mT with a frequency range of 1 to 300 Hz and is applied in a pulsed or non-pulsed fashion. Pulsed ELF-MF is intensively investigated and used in the clinic to treat delayed union bone fractures and wounds (Pesce et al., 2013). Furthermore, pulsed ELF-MF is beneficial in preclinical models of hind limb ischemia (Li et al., 2015), myocardial infarction (Yuan et al., 2010; Hao et al., 2014) and traumatic brain injury (Rasouli et al., 2012). In contrast, NP-SEMF is less studied but shows clinical promise in a rat model of Huntington’s disease (Tasset et al., 2012). Although the precise cellular mechanism activated by ELF-MF is poorly characterized, NO is induced by both pulsed ELF-MF and NP-SEMF in the brains of healthy rats (Jelenkovic et al., 2006; Bragin et al., 2015).

Because of the promising preclinical data of NO donors as a stroke therapy and the ability of NP-SEMF to induce NO production, we investigated the effect of NP-SEMF in a rat model of cerebral ischemia. We also studied the influence of NOS inhibition on NP-SEMF-induced neuroprotective effects. To our knowledge, this study is the first to show the therapeutic effect of NP-SEMF in brain ischemia and its NO-dependence.

## Materials and Methods

### Generation of NP-SEMF

**FIGURE 1:**
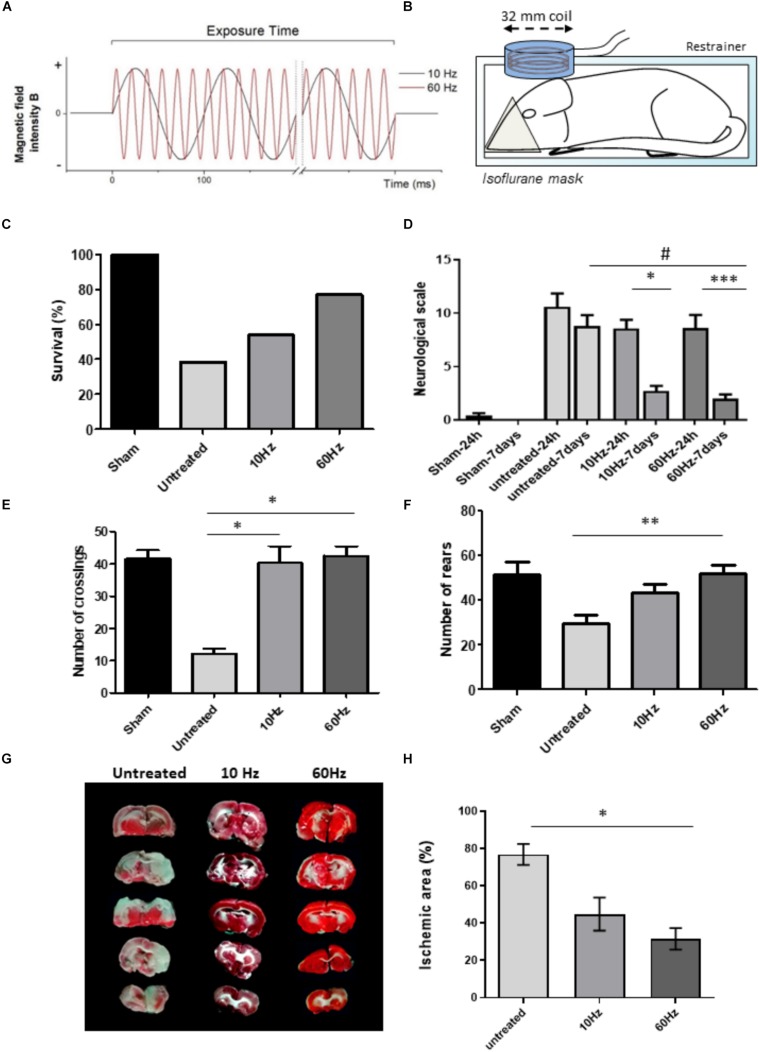
Beneficial effects of NP-SEMF in a rat model of ischemic stroke. Cerebral ischemia was induced by using the standard two-vessel occlusion (2CCAO) protocol in Wistar rats. Treated groups (10 and 60 Hz both 13.5 mT; *n* = 13/group) were repeatedly exposed to non-pulsed sinusoidal electromagnetic field (NP-SEMF) during 20 min on 4 subsequent days. The first exposure was performed 3 h after injury. **(A)** Graph showing the properties of the applied NP-SEMF. Sinusoidal magnetic waves were generated at either 10 or 60 Hz in a continuous fashion during the exposure time. **(B)** Scheme of the experimental set-up for the rat study. Rats were restricted in a plastic restrainer while receiving the anesthetic isoflurane during the NP-SEMF treatment. The plastic enclosed coil which has a diameter of 32 mm generates the NP-SEMF and is placed on top of the skull. **(C)** Survival was evaluated 7 days following stroke induction. Only 38% of the untreated animals survived 7 days after surgery while survival rates were substantially higher after NP-SEMF treatment (54% for 10 Hz and 77% for 60 Hz). **(D)** Neurological score was determined 24 h and 7 days after injury on the surviving animals. A higher score represents a higher number of deficits. *n* = 5/sham group and *n* = 13/untreated and treated groups. ^*^p ≤ 0.05 and ^∗∗∗^*p* ≤ 0.001 24 h vs. 7 days; ^#^*p* ≤ 0.001 60 Hz NP-SEMF 7 days vs. untreated 7 days post-surgery. All calculated with a Kruskall–Wallis test and Dunnett’s *post hoc* test. **(E)** Open field test was assessed before surgery (baseline) and 7 days after surgery. The frequency to cross the fourth quadrant was measured to assess the effect on locomotion. ^*^p ≤ 0.05 vs. untreated, as analyzed with a Kruskal–Wallis test with a Dunnett’s *post hoc* test. **(F)** In the open field tests, the number of rears (i.e., animal raises his forepaws) was measured as an indication of spontaneous exploratory behavior. ^∗∗^*p* ≤ 0.01 as analyzed with a Kruskal–Wallis test with a Dunnett’s *post hoc* test. **(G)** Brain slices were stained with TTC to determine infarct size in 2CCAO animals. **(H)** The damaged area as well as the whole brain area was quantified using ImageJ. For every animal, the area of affected brain tissue was quantified and calculated as % of total brain area. Next, the averages of all animals of the same experimental group were made (5 sections/brain, *n* = 8/group, data represent average ± standard deviation). TTC was performed 7 days after surgery, on animals without treatment and animals which received 10 Hz or 60 Hz NP-SEMF. ^*^p ≤ 0.05, ^∗∗∗^*p* ≤ 0.001 vs. control, as analyzed with a Kruskal–Wallis test with a Dunnett’s *post hoc* test.

Based on our previous results using NP-SEMF for pain relief (Niubó Elías et al., 2010), we used an electromagnetic field strength of 13.5 mT and a non-pulsed sinusoidal frequency of 10 and 60 Hz during a short application of 20 min. NP–SEMF was generated using a coil (ferromagnetic core radius 16 mm; wire diameter 0.20 mm; 950 turns) connected to a Magnetic Stimulator NaK-02. The NaK-02 function generator was coupled to a power amplifier (high fidelity amplifier; bandwidth 10 Hz–20 kHz; output 60 W) to generate a continuous sinusoidal current source (for detailed specifications see Supplementary Data S1). The resulting magnetic field showed a sinusoidal distribution in function of time as exemplified in Figure 1A. During the exposure time, a continuous sinusoidal current was applied without intervals, which is in contrast to pulsed ELF-MF.

### Permanent Bilateral Common Carotid Arteries Occlusion (2CCAO Model) in Rats

Cerebral ischemia was induced in Wistar rats (6–8 weeks old), in which both common carotid arteries were permanently occluded with 5-0 silk suture (Yamaguchi et al., 2005). Animals that died immediately after the procedure or demonstrated bleeding during surgery were excluded. Treated groups (10 and 60 Hz both 13.5 mT) were repeatedly exposed to NP-SEMF during 20 min on 4 subsequent days, with first exposure 3 h after injury (experimental setup, see Figure 1B). A group of sham operated animals, which were subjected to the same surgical intervention but without vessel occlusion, was also included. Survival was evaluated daily for 7 days. The open field test was performed to assess general locomotor activity (measuring the number crossing the fourth quadrant) and spontaneous exploratory activity (measuring the number of rears, thus the frequency at which the rodent stands on its hind legs). This was done before surgery (baseline level) and 7 days after surgery. Neurological outcome was scored double blindly 24 h and 7 days after surgery (Supplementary Table S1) (Menzies et al., 1992). Experimental procedures were approved by the “Scientific and Ethic Council of the CNEA.” All conducted experiments were performed according the standard biosecurity and UHasselt (BIOMED), and CNEA safety procedures.

### Staining of Brain Sections With 2,3,5-Triphenyltetrazolium Chloride (TTC)

Seven days after surgery, animals were sacrificed. Brain slices of 1 mm were made and stained for 30 min in a 2% TTC solution (Sigma, St. Louis, MO, United States). TTC stains non-damaged tissue in red, while death tissue remains white. Infarct quantification was performed on digital photographs using ImageJ (NIH, Bethesda, MD, United States).

### NO Production by HMEC-1 Cells After NP-SEMF

Human immortalized microvascular endothelial cells (HMEC-1) were seeded in 24 well plates (40,000 cells/well). 24 h later, the medium was replaced and cells were treated with NP-SEMF (13.5 mT, 20 min. 10 or 60 Hz) with or without the NOS inhibitor L-NMMA at different concentrations (1.5 mM, 150 μM, 15 μM and 1.5 μM). L-NMMA was added 15 min before NP-SEMF (A scheme of the experimental setup is provided in Supplementary Figure S2). 1 h or 24 h later, medium was collected and the amount of nitrite (a non-volatile breakdown product of NO) was measured using Griess assay (Promega, Leiden, Netherlands).

### *In vivo* Inhibition of NO After NP-SEMF Treatment

Cerebral ischemia was induced in male Wistar rats by 2CCAO. The NOS inhibitor (L-NAME) was injected intraperitoneally (20 mg/kg) (Delwing et al., 2005) once 15 min before surgery and daily 15 min before NP-SEMF treatment. Untreated animals received an intraperitoneal saline injection. Survival was monitored daily. At day 7, the rats were sacrificed and TTC staining was performed.

### Statistical Analysis

Statistical analysis was done with Graphpad Prism software (Graphpad, San Diego, CA, United States). Statistical significance was set at *p* < 0.05.

## Results

### NP-SEMF Has Beneficial Effects in a Rat Model of Stroke

Cerebral ischemia was induced in rats by permanent occlusion of the two common carotid vessels. Animals where either untreated or received NP-SEMF treatment (10 or 60 Hz both 13.5 mT; *n* = 13/group) for 20 min during 4 subsequent days. Without treatment, the surgery causes death of 62% of the animals after 7 days (Figure 1C). NP-SEMF considerably improved the survival rates by 1.4-fold for 10 Hz and by 2.0-fold for 60 Hz. The neurological scale was assessed after 24 h and 7 days post-surgery (Figure 1D). For the untreated group, no statistical differences were found between 24 h and 7 days. In contrast, in both NP-SEMF groups, a significant improvement was evident after 7 days (from 8.6 ± 1.2 to 2.7 ± 0.5 for 10 Hz, and from 8.6 ± 0.8 to 2.0 ± 1.1 for 60 Hz). Functional outcome was measured with the open field test (Figures 1E,F). NP-SEMF treatment induced a significant improvement of locomotion as shown by a rescued frequency of crossing the fourth quadrant: from 12.2 ± 1.5 (untreated) to 40.2 ± 5.2 (10 Hz) and 42.5 ± 3.0 (60 Hz). The number of rears was significantly increased for the 60 Hz treated group (29.6 ± 3.8 in untreated animals versus 51.7 ± 4.1 in 60 Hz).

During NP-SEMF, animals are anesthetized with isoflurane and placed in a restrainer. To rule out any effect of this procedure, we studied animals (with 2CCAO) placed in the restrainer but without switching on the magnetic flux ( = sham exposed animals) (Supplementary Figure S1). No amelioration in survival nor neurological outcome were observed.

NP-SEMF reduced the severity of the ischemic brain damage(Figures 1G,H). TTC staining 7 days after injury revealed that untreated animals displayed large white infarcted areas in both the cortex and the striatum as quantified with ImageJ. The infarcted brain area was reduced by more than 50% after 60 Hz NP-SEMF treatment and the remaining lesions were mainly located in the striatum. The percentage of ischemic brain area in control animals was significantly reduced from 76.9 ± 5.6% toward 31.5 ± 5.8% in animals treated with 60 Hz. 10 Hz application did lead to a slight reduction in lesion size, although this difference was not statistically significant.

### Inhibition of NOS Decreased NP-SEMF-Induced NO Production *in vitro*

**FIGURE 2:**
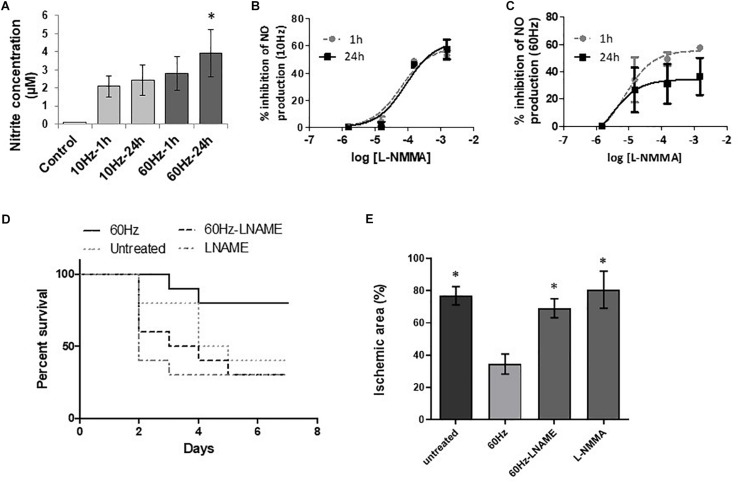
Involvement of NO as key mediator of the beneficial effects of NP-SEMF. **(A)** Nitrite concentration (as an indirect measure for the NO concentration) induced by NP-SEMF was measured by Griess reaction, 1 h or 24 h after treatment of NP-SEMF for 20 min (13.5 mT at 10 or 60 Hz). Values are expressed as mean ± SD, *n* = 4, ^*^p ≤ 0.05, as calculated with Kruskal-Wallis ANOVA test with a Dunnett’s *post hoc* test. **(B)** Inhibitor effect of L-NMMA (1.5 mM, 150 μM, 15 μM, 1.5 μM) on NO production by HMEC-1 subjected to 10 Hz NP-SEMF for 20 min, *n* = 4. NO production was measured 1 h or 24 h after treatment. **(C)** Inhibitor effect of L-NMMA on NO release by HMEC-1 subjected to 60 Hz NP-SEMF. NO production was measured 1 h or 24 h after treatment, *n* = 4. **(D)** Rats were subjected to 2CCAO and received either no treatment, L-NAME injections, 60 Hz NP-SEMF or combination of L-NAME and NP-SEMF. L-NAME was injected at a dose of 20 mg/kg, 15 min before surgery and before each NP-SEMF treatment (13.5 mT for 20 min at 10 or 60 Hz). Kaplan-Meier graph shows the survival rates of these four groups during 7 days after surgery (*n* = 10/group). The percentage of surviving animals was higher after NP-SEMF treatment. L-NAME injection inhibited the beneficial effect of NP-SEMF on survival. **(E)** The ischemic area in the brain of the surviving rats was measured by TTC staining 7 days after surgery and calculated as ischemic area (in % of total area). Treatment of L-NAME obliterates the neuroprotective effect of NP-SEMF. *n* = 4/group, ^*^p ≤ 0.05 vs. 60 Hz, according to a Kruskal–Wallis ANOVA test with a Dunnett’s *post hoc* test.

To verify that NP-SEMF acts via NO, the NO production was indirectly measured with the Griess assay in HMEC-1 cells (Figure 2A). Nitrite concentration (and thus NO) was significantly increased 24 h after 60 Hz NP-SEMF (3.9 ± 1.3 μM in 60 Hz vs. control). The involvement of NOS was studied using the general NOS inhibitor L-NMMA, which was able to dose-dependently inhibit the NP-SEMF-triggered NO production (Figures 2B,C). The IC_50_ for 10 Hz was estimated 59.88 μM after 1 h and 85.53 μM after 24 h; The IC_50_ for 60 Hz was 7.3 μM after 1 h and 2.7 μM after 24 h.

### NOS-Derived NO Was Essential for NP-SEMF-Effectivity *in vivo*

To prove the involvement of NO in the neuroprotective action of NP-SEMF after ischemic stroke, the general NOS blocker L-NAME was injected in 2CCAO rats to inhibit NO formation. The beneficial effect of NP-SEMF at 60 Hz on survival 7 days after ischemic stroke disappeared when NO-synthesis was blocked (Figure 2D). Inhibition of NOS with L-NAME inhibited the protective effect of NP-SEMF on tissue damage as the lesion size (in % of total area) was significantly increased (NP-SEMF+L-NAME 69.1 ± 3.4 vs. NP-SEMF alone 34.5 ± 6.2). The tissue damage in animals treated with both NP-SEMF and L-NAME were similar in size compared to the untreated group (Figure 2E). These results indicate that NO is a key signaling molecule by which NP-SEMF protects the brain from tissue damage after ischemic stroke.

## Discussion

As several studies have demonstrated the ability of ELF-MF to alter NO production and since NO has been suggested to be beneficial in stroke, we explored the use of NP-SEMF in a rat model of severe cerebral ischemic stroke. Daily applications of 20 min of NP-SEMF (10 and 60 Hz) significantly improved survival, locomotion, and neurological outcome. The lesion size was dramatically reduced in rats treated with 60 Hz NP-SEMF. Our study confirmed that NO is the key player in this beneficial effect of NP-SEMF. NP-SEMF increased NO production in HMEC-1 cells, which was inhibited by the NOS-inhibitor L-NMMA. Moreover, the NOS blocker L-NAME reversed the beneficial effects of NP-SEMF in rats subjected to 2CCAO. This is in line with previous findings which showed that L-NAME prevents blood flow induction by pulsed ELF-MF in healthy rat brains (Bragin et al., 2015). Accordingly, L-NAME also reduced the angiogenic effects of endothelial cells induced by pulsed ELF-MF (Li et al., 2015).

We postulate that NP-SEMF is effective by inducing NO. In the acute phases of stroke, relative NO depletion occurs, leading to a reduction of cerebral blood flow (CBF) and the development of secondary damage. Therefore, increasing post-ischemic NO content to augment CBF toward the lesion is considered to be a promising treatment strategy for ischemic stroke. Indeed, numerous animal studies demonstrate the efficacy of NO donors or inhaled gaseous NO as an ischemic stroke therapy (Garry et al., 2015). Nevertheless, in the clinic, application of the NO precursor L-arginine or the NO donor glyceryl trinitrate has no beneficial effects in ischemic stroke (Bath et al., 2000), while other strategies based on NO (such as NO inhalation) are still in the experimental stage (Terpolilli et al., 2016). It is highly likely that NP-SEMF has a similar effect as NO donors or gaseous NO. An additional advantage of NP-SEMF is that it locally induces NO production in regions with interrupted blood flow, whereas gaseous NO or NO donors are administered systemically so might not reach the damaged regions and may provoke unwanted side effects (Garry et al., 2015). Possibly, NP-SEMF also activates other pathways independent of NO.

The mechanism by which NP-SEMF stimulates NO production remains to be investigated in detail. Previous studies on healthy brains show enhanced NO levels in the brain but the underlying mechanism could not be revealed. NP-SEMF induces eNOS and iNOS expression in human keratinocytes (Patruno et al., 2010), while pulsed ELF-MF enhances phosphorylation of eNOS in a mouse hindlimb ischemia model (Li et al., 2015).

Taken together, our data clearly show the high potential of NP-SEMF to improve neurological outcome, lesion size and survival in a rat model of stroke. The fact that an electromagnetic field of low frequency during short time frames causes such a tremendous improvement sparks hope to translate this new “electroceutical” into the clinic.

## Data Availability

The datasets generated for this study are available on request to the corresponding authors.

## Ethics Statement

This study was carried out in accordance with the regulations of Scientific and Ethic Council of the CNEA (Centro Nacional de Electromagnetismo Aplicado), Santiago de Cuba, Cuba. The protocol was approved by the “Scientific and Ethic Council of the CNEA” (Centro Nacional de Electromagnetismo Aplicado), Santiago de Cuba, Cuba.

## Author Contributions

LF carried out the *in vivo* experiments and wrote the first draft of the manuscript. LF, HK, and AB carried out the *in vitro* experiments, histology, and prepared the figures. LF, MC, AB, and BB were involved in data interpretation. MC designed the NP-SEMF equipment. FG was responsible for device calibration. LF, AB, BB, HK, MC, RM, FG, OS, IL, and J-MR contributed to the project design and reviewed the manuscript. All authors who contributed to manuscript revision, read, and approved the submitted version.

## Conflict of Interest Statement

The authors declare that the research was conducted in the absence of any commercial or financial relationships that could be construed as a potential conflict of interest.
