# Supplementary material for: Non-pulsed Sinusoidal Electromagnetic Field Rescues Animals From Severe Ischemic Stroke via NO Activation
Source: Front Neurosci. 2019 Jun 19;13:561. doi: 10.3389/fnins.2019.00561 (PMC6593085; doi:10.3389/fnins.2019.00561)
Supplement: Supplementary file 1 [file Data_Sheet_1.PDF]

Supplemental data S1:

Specifications and validation of the NP-SEMF generator

NP –SEMFs were generated using a coil (ferromagnetic core radius 16 mm; wire diameter 0.20 mm; 950 turns) connected to a Magnetic Stimulator NaK-02 and power amplifier. NaK-02 is a function generator manufactured by the Centro Nacional de Electromagnetismo Aplicado (CNEA), Cuba, registered under the File Number I0350015041150 and the Code Number 73BWK by the CCEEM and the MINSAP, and certified for fulfilment with parametric and safety requirements by two accredited laboratories: Oficina Territorial de Normalización, Santiago de Cuba, and Centro de Estudios de Tropicalización, Havana, Cuba. Furthermore, it is certified by the CNEA Magnetic Characterization Laboratory. The NaK-02 function generator was coupled to a power amplifier (high fidelity amplifier; bandwidth 10Hz-20 kHz; output 60 W) manufactured at CNEA to generate a continuous sinusoidal current source. The magnetic field generated under the coil was measured using a calibrated model 5180 F.W, Bell gauss meter (Pacific Scientific, OECO, USA) of which the probe was positioned using a micrometric positioning system. The resulting magnetic field has a sinusoidal distribution in function of time. During the exposure time a continuous sinusoidal current was applied without intervals, which is in contrast to pulsed magnetic stimulation. You can find the patent of this simulation device on:
https://worldwide.espacenet.com/publicationDetails/biblio?FT=D&date=20040123&DB=&locale=&CC=CU&NR=22896A1&KC=A1&ND=1;
(Cuban patent No. CU22896A1, application number CU20010001A).

The magnetic field generated by a coil is not homogenously distributed in space and therefore the resulting magnetic field (B) reaching the experimental set-up needed to be verified. As the various experiments required different working volumes, this measurement was performed for every recipient or exposure system used and their corresponding working volume (Table 1). The minimum, maximum and the mean intensity of the magnetic field (respectively Bmin, Bmax and Bmean) were determined after measuring in 100 points randomly distributed in the working volume. The electric current intensity through the coil was adjusted in order to obtain a comparable mean value of the magnetic induction in all exposure systems.

Table 1: The intensity of the magnetic field in the various exposure systems

**B(mT)**	**Rat Brain**	**24 well (NO assay)**
**Bmin ± SD**	2.12 ± 0.45	2.12 ± 0.85
**Bmean ± SD**	3.25 ± 0.69	3.24 ± 1.30
**Bmax ± SD**	4.38 ± 0.92	4.37 ± 1.75
