# Supplementary figures and images for: Non-pulsed Sinusoidal Electromagnetic Field Rescues Animals From Severe Ischemic Stroke via NO Activation
Source: Front Neurosci. 2019 Jun 19;13:561. doi: 10.3389/fnins.2019.00561 (PMC6593085; doi:10.3389/fnins.2019.00561)

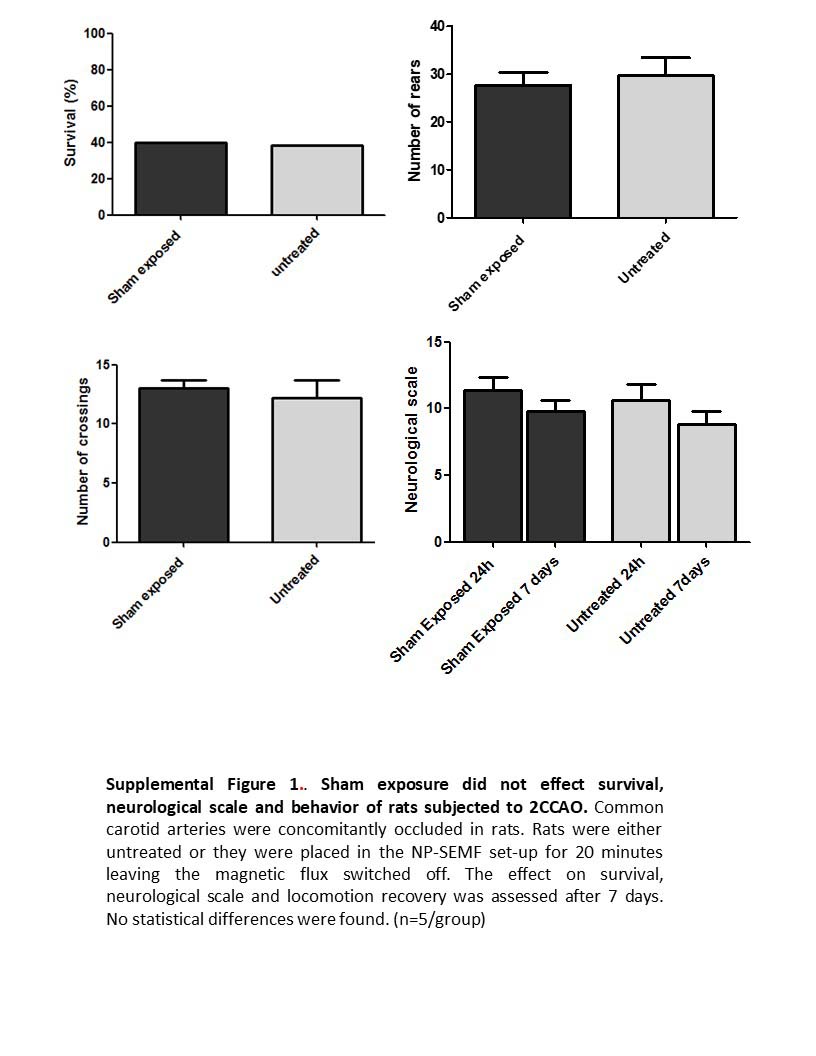

Supplement: Supplementary file 2 [file Image_1.JPEG]

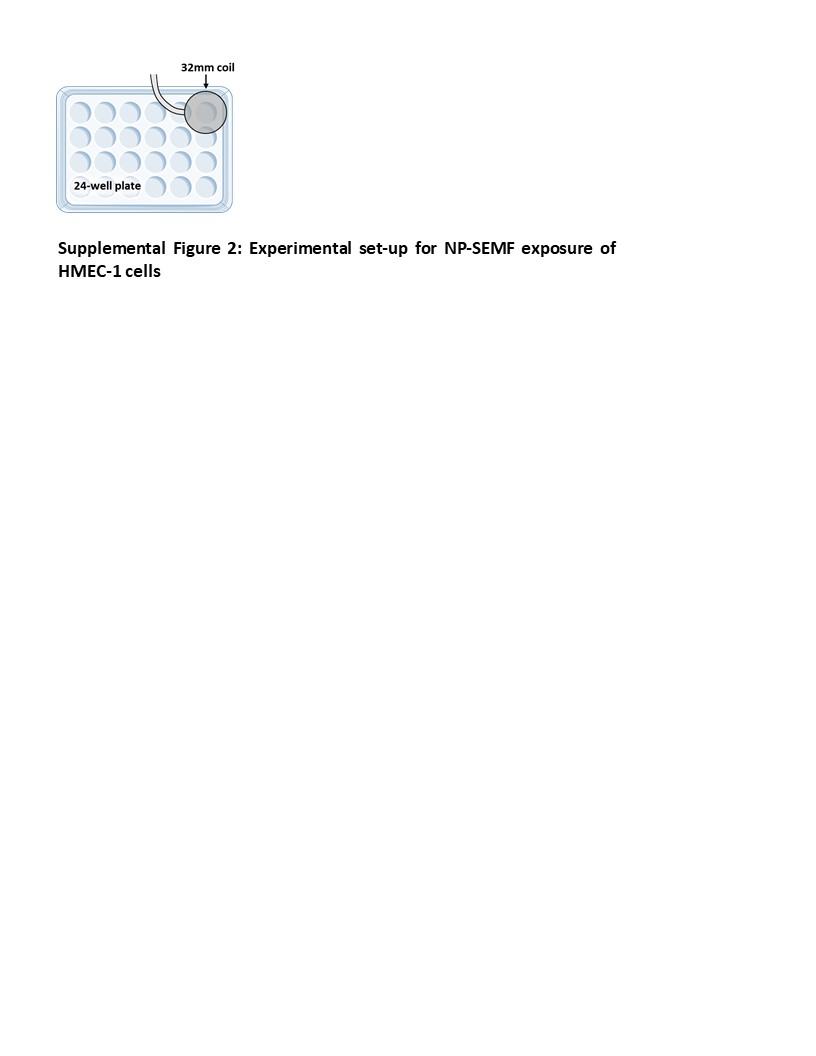

Supplement: Supplementary file 3 [file Image_2.JPEG]
