# Supplementary material for: Non-pulsed Sinusoidal Electromagnetic Field Rescues Animals From Severe Ischemic Stroke via NO Activation
Source: Front Neurosci. 2019 Jun 19;13:561. doi: 10.3389/fnins.2019.00561 (PMC6593085; doi:10.3389/fnins.2019.00561)
Supplement: Supplementary file 4 [file Table_1.docx]

**Supplementary Table 1: Neurological Scale**

| **Signs** | **Points** | **Conditions** |
| --- | --- | --- |
| **Ptosis** | 1 | Semi-closed left eye |
|  | 2 | Semi-closed right eye |
|  | 3 | Completely closed left eye |
|  | 4 | Completely closed right eye |
| **Bristling** | 1 | Pilo-erection |
| **Hypotony** | Decreased muscle tone, grip strength and / or motor function: | |
|  | 1 | Left, front or rear leg |
|  | 2 | Right, front or rear leg |
| **Hyporeflexy** | Reduction of the flexor reflex to stretching or pinching of members: | |
|  | 1 | Left, front or rear leg |
|  | 2 | Right, front or rear leg |
| **Posture** | 1 | Head tilted to the left, C-shaped body |
|  | 2 | Head tilted to the right; inverted C-shaped body |
| **March** | 1 | Slow |
|  | 2 | None |
| **Turns** | 1 | Move up to the left (Contralateral) |
|  | 2 | A circular motion to the right (Ipsilateral) |
|  | 3 | Left turns |
|  | 4 | Right turns |
